# First night effect alters occipital brain connectivity in horses

**DOI:** 10.1038/s41598-025-14830-2

**Published:** 2025-08-17

**Authors:** Jürgen Bergeler, Astrid Liske-Schmitz, Thomas Schmitz, Nora Vanessa de Camp

**Affiliations:** 1 Petesys UG (ltd), Hauptstraße 3a, Haseloff, Mühlenfließ, 14823 Brandenburg Germany; 2 Institute of Animal Welfare, Animal Behavior and Laboratory Animal Science, Free University, Berlin, Germany; 3 Equine rehabilitation center, Gut Marggraffshof GmbH, Marggraffshof 2, Marggraffshof , 14532 Brandenburg Germany

**Keywords:** Sleep, Neurophysiology

## Abstract

The First Night Effect is a phenomenon whereby sleep duration and quality are compromised in unfamiliar environments or situations. Horses are often transported to new locations, such as sporting events. We wanted to know if the First Night Effect is also detectable in horses in two different populations. To investigate this, we compared five horses from a professional sports horse barn that are regularly used in competitions, with six horses from a breeding barn, that are less frequently transferred to unfamiliar places. Despite the significant differences observed in electroencephalography (EEG) patterns and behavior between the two horse populations, we were able to identify EEG changes indicative of the First Night Effect. These changes were most apparent in the delta band at the occipital position of the brain, indicating fluctuations in sleep-wake dynamics and consciousness. Furthermore, in this study, EEG data outperformed behavioral data in the detection of the First Night Effect, indicating the usefulness of EEG measurements for monitoring welfare or even assessing stress and pain.

## Introduction

The First Night Effect describes a compromised sleep quality, especially in novel environments, such as a sleep laboratory room in human studies^1,2^. New insights suggest that an unfamiliar environment is not the only exclusive trigger for the First Night Effect, but also the EEG (electroencephalography) application procedure itself and possibly an associated higher vigilance state^3^. Tamaki et al.^4^ found an asymmetry in hemispheric vigilance in humans, indicating that at least one hemisphere serves as a “night watch” survival strategy in unfamiliar environments. Unilateral sleep has been described in marine mammals, birds, and reptiles^5^. The First Night Effect most often impacts the slow wave activity of the EEG^4^. An increase in the heart rate (representing the autonomous nervous system) leads, for example, to an increase in beta band EEG activity in relation to the slower delta band activity and is associated with the First Night Effect^6^. To limit the adverse side effects of the First Night Effect on sleep studies, it is recommended to use an accommodation night prior to the start of the experimental sleep recordings^7^.

The First Night Effect has been described in different animal species. A decrease in REM sleep and rumination has been shown in dromedary camels^8^. In contrast, the First Night Effect leads to a higher amount of REM sleep in cats^9^. The First Night Effect in dogs afternoon sleep influenced sleep latency and sleep efficiency but not the amount of REM or NREM sleep^10^. We found no studies on the First Night Effect in horses.

Horse sleep EEG was described in detail by Williams et al.^11^. Drowsiness is characterized by 20 Hz beta band activity as well as 4 Hz delta band activity, and slow wave sleep consists of background activity from 1 to 4 Hz and transient events, such as K-complexes, sleep spindles, and vertex transients. REM sleep occurred while standing or lying and consisted of 20–30 Hz activity and rhythmic 4 Hz activity^11^.

The sleeping behavior of horses is polyphasic, with fragmented sleep cycles across 24 h^12^. Total sleep time is only about 50% of human sleep time, but the relative amount of non-REM sleep (77,5%) and REM sleep are similar to human (82% and 17%)^12^. Although sleep cycles are also apparent during the daytime, most sleep occurs at night in horses^13^.

A study from the University Center Hartpury (UK) showed that horses sleeping behavior is altered by seasonal management practices^12^. Ruckebusch^14^ also showed the effect of a novel environment on the sleeping behavior of horses.

A review on horse sleep and its implications for welfare was provided by Fewings and Greening^15^. They raised the question of whether training (physical exercise) has an impact on sleep behavior. For humans, most studies have found extended sleep duration and quality after physical exercise, but only for certain age groups^16^, and especially for populations suffering from disease. Hence, sleep duration may be a problematic welfare indicator in animals.

Other welfare-associated factors that can impact sleeping behavior include pain, social stress, light, noise, and a novel environment^12^.

The goal of this study was to extract the essence of the First Night Effect in the EEG patterns of horses. Therefore, we compared two populations. A population of sport horses with some habituation to novel environments and frequent travel. And a non-sport horse population with less frequent changes in the resting location.

## Methods and materials

All procedures were approved by the local ethics committees (#2340-11-2019, LAVG Brandenburg, general animal welfare and # 81.02.05.10.02.03, LANUV, North Rhine Westphalia), and followed the European and the German national regulations (European Communities Council Directive, 86/609/ECC; Tierschutzgesetz).

All animal procedures were performed in accordance with the [Freie Universität Berlin] animal care committee’s regulations. The study is reported in accordance with ARRIVE guidelines. Due to minimal stress, no pain and unchanged routines, the procedure was not classified as animal testing. The breeding horse-population belongs to the equine rehabilitation center Marggraffshof (Germany, Brandenburg) and the sport horse population to the DOKR (German Olympic Committee for Equestrian Sports e. V., Warendorf, Germany) and the FN (German Equestrian Federation, Warendorf, Germany).

EEG measurements were taken in the barn, and each horse was housed in an individual box (4 × 4 m, some extremely large Shire horses had larger boxes, relative to their size) during the night. During the day, horses underwent training and/or free movement on pasture or paddock. The normal routines were not changed for the EEG measurements; horses were handled, trained, and fed as usual. The horses were not restrained and moved freely in the horse box with the EEG system. The boxes were lined with straw or shavings, and there was visual contact and odor contact through the bars between the horses.

Because sleep patterns in the brain are broadly linked to the delta band, we used a telemetric full-band DC EEG recording system. It has been previously shown that the delta band appearance is altered with AC coupled amplification systems^17^. The telemetric full-band DC EEG system with eight recording electrodes (sampling rate 1000/s) was placed on the neck of the horses right behind the ears, in a small key pocket attached to the neckband of the halter. The antennae and cameras were placed inside the box at a higher position, out of the horse’s range. Camera recordings were used to verify whether the horses were drowsy but standing or if they were lying down to sleep.

The electrodes were placed at the occipital, parietal, temporal, and frontal positions on the right and left hemispheres, respectively. The positions were adapted from the study by Williams et al.^11^ (O_1_,O_2_,P_4_,P_3_,C_4_,C_3_,F_4_,F_3_). The reference electrode was placed on the frontal part of the nose, and the ground electrode was placed caudal to the occipital bone. The procedure took approximately 10 min in the horse’s home box with the assistance of a familiar person. All horses included in this study were familiar with the handling procedures, and no sedation was used. Head-shy horses were excluded from the study.

**Fig. 1:**
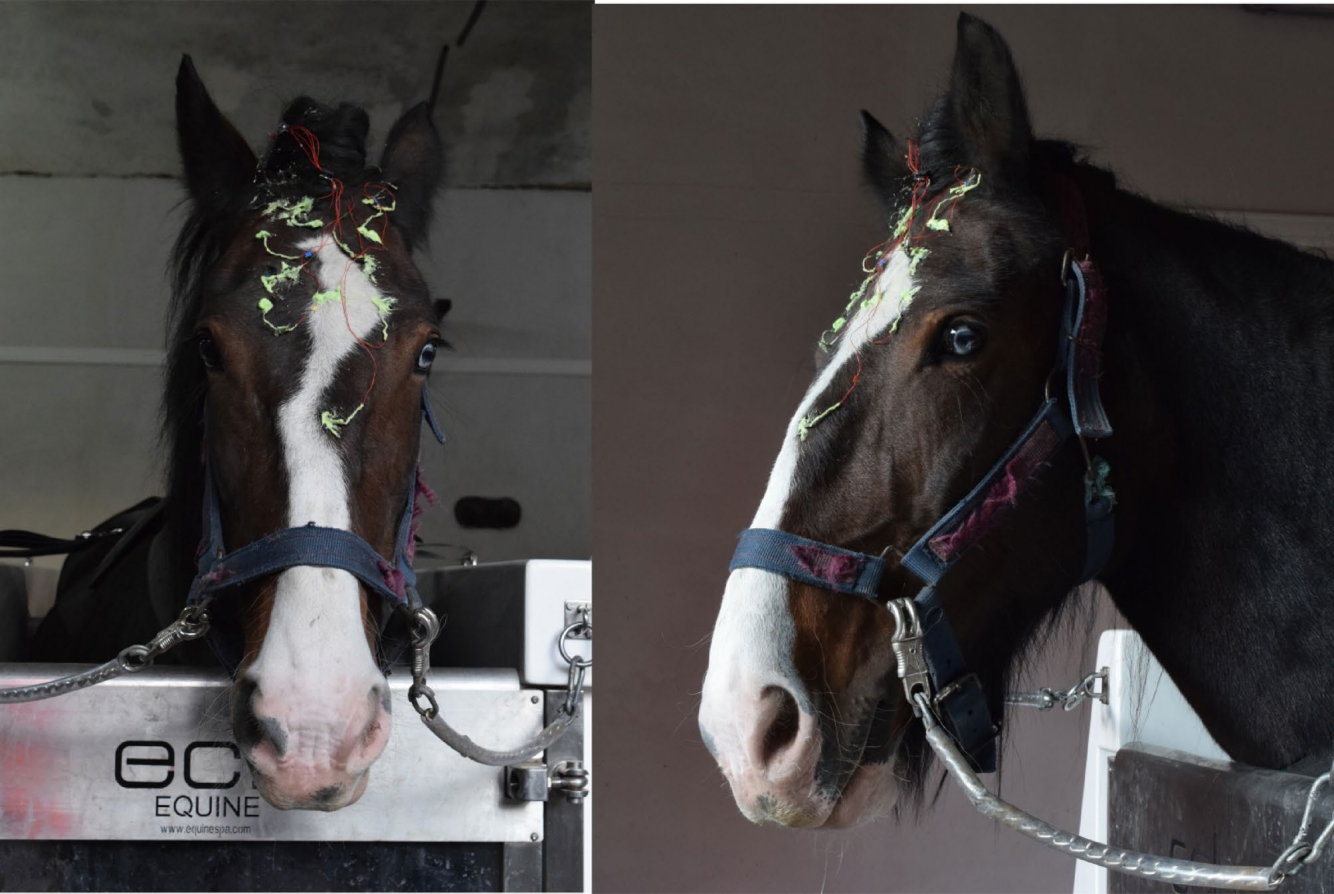
Horse with EEG system and electrodes. The green cover above the electrodes is a silicone, meant to protect them. The telemetric amplification system is placed in a pocket on the neck band of the halter.

The electrodes consisted of a light-cured conductive polymer material, applied on the unshaved skin and cured within 10–20 s with a dental blue light^18^. Before electrode application, the skin/hair was gently cleaned with fine-grained sandpaper. This mechanical cleaning technique is similar to the natural technique used by horses, is widely accepted and establishes good conductive contact between the electrode and skin. The diameter of one electrode was 5 mm. The polymer electrode was attached to the EEG amplifier via 5 cm long conductive textile cables and crimped to an insulated copper wire. The whole cable length was approximately 30 cm, depending on the horse (size of the head, shire horse bigger than warmblood/thoroughbred) and the EEG position (occipital cables shorter than frontal cables). The electrodes were protected using green silicone rubber (Body Double Silk, Smooth On Inc., USA) (Fig. 1).

Data acquisition was performed using a miniaturized transceiver developed by us, which recorded the EEG data metrologically and transmitted it to a receiver unit via the 2.4 GHz IMS band (Industrial, Scientific, and Medical). Direct coupling of the sensitive amplifier to the signal source enabled 8-channel DC acquisition of the EEG signals from 10 to 50µV in a voltage range of +/- 150mV. After analog processing, the signals were digitized at a sampling rate of 1000 samples per second and then processed for transmission. The entire digital processing was performed by a microcontroller from Nordic (nRF52822), which also contained a transceiver. Owing to the high integration density of the microcontroller and the resulting extremely small size (3 × 4 cm), the entire transceiver could be made light and small. In addition to the transceiver, a receiver unit was developed that stored the received data on a laptop via a USB connection. The largest and heaviest component of the transceiver was the power supply, which had to ensure operation for at least 12 h. As the use of lithium batteries seemed too risky from a fire safety perspective given the situation in the barn, we used standard alkaline manganese batteries of size “AA”.

A total of 12 horses were measured, 6 sport competition horses (eventing, warmblood horses with a high proportion of Thoroughbred, one mare, five geldings) and six non-sport horses (five Shire horses, one Knabstrupper, three mares, two geldings, one stallion).

The sport horses underwent high-intensity daily training and were used regularly for competition events. The breeding and leisure horses underwent light daily training. Owing to these differences, feeding patterns differed among horse populations. The hay feeding regimen was similar: all horses received their afternoon/night hay after the electrode fixation procedure.

Similar to other studies^12,14^, we classified behavior as foraging, drowsiness, and recumbency (lying down). All other behavioral outcomes were subsumed as “other”. Behavioral observation was initiated in the evening after electrode placement until the next morning.

Video analysis was performed by viewing. Feeding was identified by the ingestion of hay, active foraging, and chewing. Drowsiness was identified by a lowered neck and head position, no feeding, but still standing. Recumbency was identified by lying down.

We lost some camera data due to −20 °C temperatures during very cold recording nights. Therefore, we were only able to show behavioral data for four horses in the sport horse guest-box condition.

All horses were recorded for one night under two different conditions:

the home box (the usual box, which the horse was familiar with) and a guest box (to which the horse was transferred for only one night).

The home box was permanent for the non-sport horses. The sport horses also had a home box, but this home box was changed approximately on a monthly basis due to management procedures. The guest boxes were located at the same respective property as the home box, and the horses were led into the new box without the need for transportation by vehicle. Horses had at least two box neighbors in both conditions (home box and guest box). However, the box neighbors of the home box were not moved, so there were unfamiliar neighbors in the guest boxes.

The sport horses had a mean age of 10.5 years with a standard deviation of 5.5 years. The non-sport horses had a mean age of 10.2 years with a standard deviation of 4.2 years. The electrode-fixation procedure took place in the afternoon before the hay-feeding time of each session both for home box and the guest box. High quality EEG sequences were selected from night-time recordings after feeding.

A habituation period of four hours was allowed for each horse to acclimatize to wearing the equipment.

Data analysis was conducted using MATLAB 2023a (from MathWorks). EEG segments without artifacts (such as muscle interference or extended line noise) were chosen for analysis.

EEG raw data were filtered using digital Butterworth filters with a custom written MATLAB script. The filter was designed using the butter function with a 3rd order. We calculated the normalized cutoff frequency (Wn) for EEG bands delta [0–4 Hz], theta [4–8 Hz], alpha [8–13 Hz], beta [13–30 Hz], gamma [30–120 Hz]. Wn is a number between 0 and 1, where 1 corresponds to the Nyquist frequency which is half the sampling rate (in this case, 500 Hz).

The numerator and denominator values (IIR filter)obtained from the butter function butter, were used with the MATLAB function filtfilt to filter the EEG data. For the delta EEG band, a low-pass filter was applied. All other EEG frequency bands were extracted using a bandpass filter design.

To calculate correlations between electrode positions we used the MATLAB toolbox Measure of Effect Size (Harald Hentschke, (2025) hhentschke/measures-of-effect-size-toolbox (https://github.com/hhentschke/measures-of-effect-size-toolbox), GitHub^19^,. We utilized the function “mes” with the specifications “rbcorr” and “nBoot” (bootstrap, 3000 iterations, number of bootstrap samples to draw). Correlations were calculated for each band separately and for 28 electrode combinations (refer to Table 1). The median correlation was calculated for each group of horses under the conditions of the guest box and home box, respectively.

**Table 1:** Measure of effect size, channel identification, electrode pairs.

correlation analysis number	compared EEG channels	corresponding area of electrodes	abbreviation
1	1/2	frontal right/temporal right	fr/tr
2	1/3	frontal right/parietal right	fr/pr
3	1/4	frontal right/occipital right	fr/or
4	1/5	frontal right/occipital left	fr/ol
5	1/6	frontal right/parietal left	fr/pl
6	1/7	frontal right/temporal left	fr/tl
7	1/8	frontal right/frontal left	fr/fl
8	2/3	temporal right/parietal right	tr/pr
9	2/4	temporal right/occipital right	tr/or
10	2/5	temporal right/occipital left	tr/ol
11	2/6	temporal right/parietal left	tr/pl
12	2/7	temporal right/temporal left	tr/tl
13	2/8	temporal right/frontal left	tr/fl
14	3/4	parietal right//occipital right	pr/or
15	3/5	parietal right/occipital left	pr/ol
16	3/6	parietal right/parietal left	pr/pl
17	3/7	parietal right/temporal left	pr/tl
18	3/8	parietal right/frontal left	pr/fl
19	4/5	occipital right/occipital left	or/ol
20	4/6	occipital right/parietal left	or/pl
21	4/7	occipital right/temporal left	or/tl
22	4/8	occipital right/frontal left	or/fl
23	5/6	occipital left/parietal left	ol/pl
24	5/7	occipital left/temporal left	ol/tl
25	5/8	occipital left/frontal left	ol/fl
26	6/7	parietal left/temporal left	pl/tl
27	6/8	parietal left/frontal left	pl/fl
28	7/8	temporal left/frontal left	tl/fl

Measure of effect size, channel identification.

Coherence (correlation in the frequency domain) between electrode pairs was calculated using the MATLAB function mscohere (magnitude-squared coherence) with the parameters window = hanning (2000), noverlap = 100, nfft = 1000 and fs (sampling rate) = 1000. To evaluate cross-frequency phenomena, we compared the delta band of one EEG channel to the raw EEG data of another channel. All coherence results for all horses were organized in a three dimensional array with the first dimension representing the frequency band, the second dimension representing the channel pair and the third dimension representing individual horses. The median was calculated along the third dimension of the array, providing a median coherence value for each calculated coherence value across different horses. For example the median coherence between the delta range and six Hz in EEG Channel five across all five non-sport horses. Separate median values were calculated for the conditions of home and guest box for both horse-populations. Only coherence values above 0.8 (on a scale from 0 to 1) were considered.

The cross correlation was calculated using the MATLAB function xcorr. The highest xcorr values were measured at zero delay for all datasets, hence xcorr at zero delay was used for further analysis. This could also be formally described as a simple correlation. However, since we used the xcorr function in MATLAB, we will stick with xcorr for correctness. To assess intra- or interhemispheric correlations seven electrode groups have been established: H1, H2, H3, H4, V1, V2 and V3. H represents horizontal electrode pairs (interhemispheric). The lower the number, the closer the connection between the electrodes. For example, H1 consists of electrode pairs like occipital right and left or frontal right and left. H2 includes pairs like frontal right and temporal left (diagonal grade one). H3 consists of pairs like frontal right and parietal left (diagonal grade 2) and so on. The V group represents vertical electrode connections (intrahemispheric). V1 is the shortest possible connection, for example frontal right and temporal right. V2 includes pairs like frontal right and parietal right. For each horse, the normalized number of xcorr results above 0.8 was calculated for each EEG band. The basis for normalization was the maximum number of possible electrode pairs per group H1-V3. For example, V3 includes two possible channel combinations: frontal right and occipital right as well as frontal left and occipital left. If both channel combinations have xcorr values equal to or above 0.8, then the normalized value is 1. Next, the difference between the home box and guest box for each frequency band and horse was calculated. The null hypothesis, that the difference values have the same distribution for the sport horses and for the non-sport horses was then tested using a non-parametric Wilcoxon ranksum test (Table 2).

**Table 2:** Wilcoxon ranksum test Null hypothesis: same distribution of difference values of normalized amount of cross correlation between home box and guest box..

group	h	p
Theta		
H1	0	0.09
H2	0	0.05
H3	1	0.03
H4	0	0.24
V1	0	0.53
V2	1	0.04
V3	0	0.65
Alpha		
H1	0	0.09
H2	1	0.03
H3	1	0.03
H4	0	0.09
V1	1	0.004
V2	1	0.03
V3	1	0.03
Beta		
H1	1	0.01
H2	1	0.004
H3	0	0.09
H4	0	0.09
V1	1	0.005
V2	1	0.048
V3	0	0.09
Gamma		
H1	0	0.09
H2	1	0.004
H3	1	0.03
H4	1	0.03
V1	1	0.004
V2	0	0.07
V3	1	0.03

If h = 1 the null hypothesis is rejected.

Camera data from the Seissiger Wildkamera S358E, manufactured by Anton Seissiger GmbH in Germany were visually analyzed to distinguish between active behavior, drowsiness and lying down during sleep phases. EEG phases during drowsiness were specifically selected for further analysis, as some horses in each population did not lie down during the data-acquisition period. Feeding behavior was recognized by the ingestion of hay, active foraging, and chewing. Drowsiness was identified by a lowered neck and head position, absence of feeding but still standing. Recumbency was noted by the horse lying down.

## Results

### Behavioral data and video recordings

**Fig. 2:**
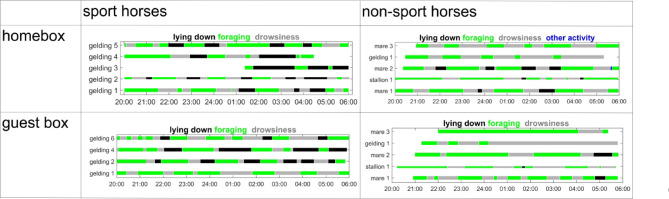
Sleep-associated behavior was observed in both horse populations, both in the home box and in the guest box. Lying down was indicated in black, feeding in green and drowsiness in grey. Due to extremely low night temperatures, we lost some data for the sport horses.

The video analysis revealed that most horses lay down with the EEG system and exhibited normal behavior in their home box. There was no statistically significant difference in the amount of feeding, lying down and drowsiness at night between the home box and the guest box (Fig. 2). However, sport horses tended to lie down for longer periods of time than non-sport horses, although this effect was only statistically significant in the home box (MATLAB Wilcoxon ranksum test, alpha 5%, p-value 0.016). Drowsiness and feeding occurred in all animals at alternating intervals of approximately the same duration. Lying down was particularly common after midnight with sport horses showing a tendency towards longer lying times (Fig. 2).

### EEG analysis: correlation

**Fig. 3:**
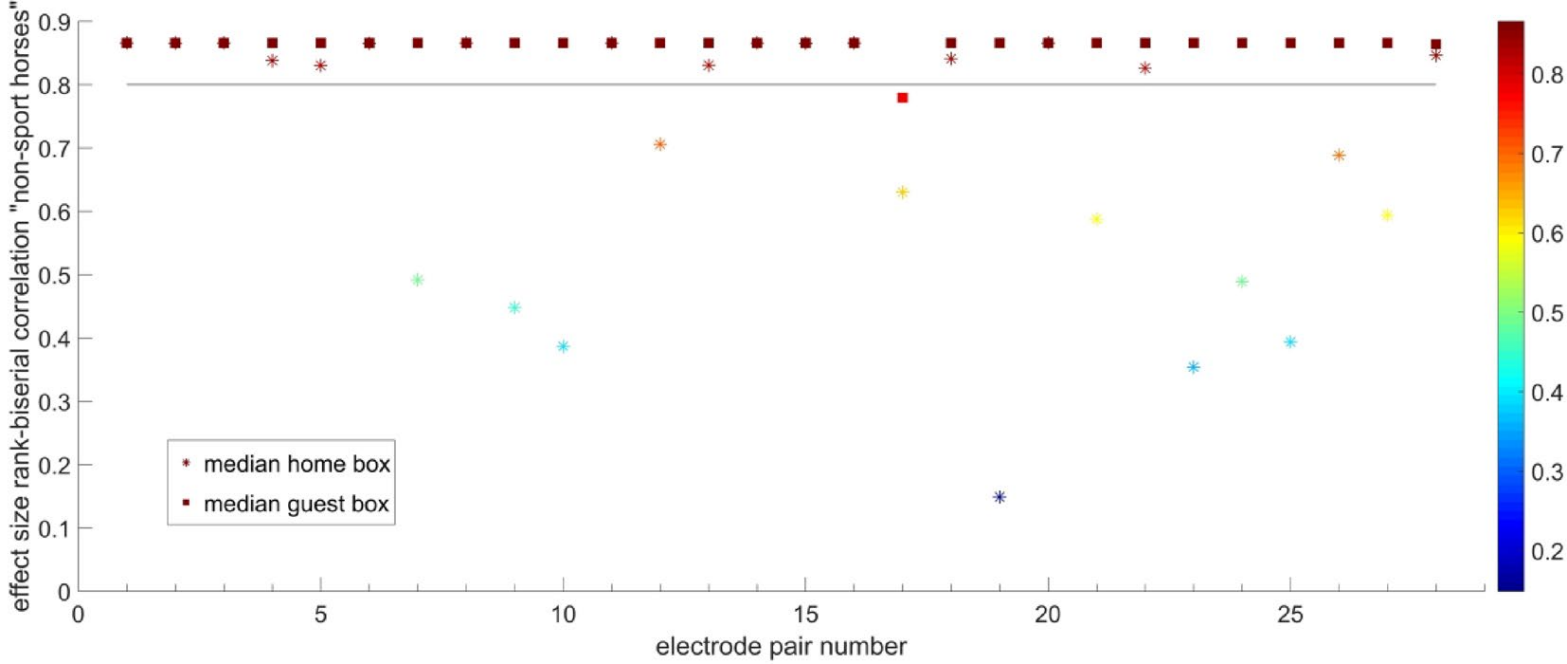
Effect size biserial rank correlation between electrode pairs for non-sport horses. Electrode pair 19 (occipital right and left, shows high correlation (threshold 0.8) in the guest box and low correlation for the home box.

**Fig. 4:**
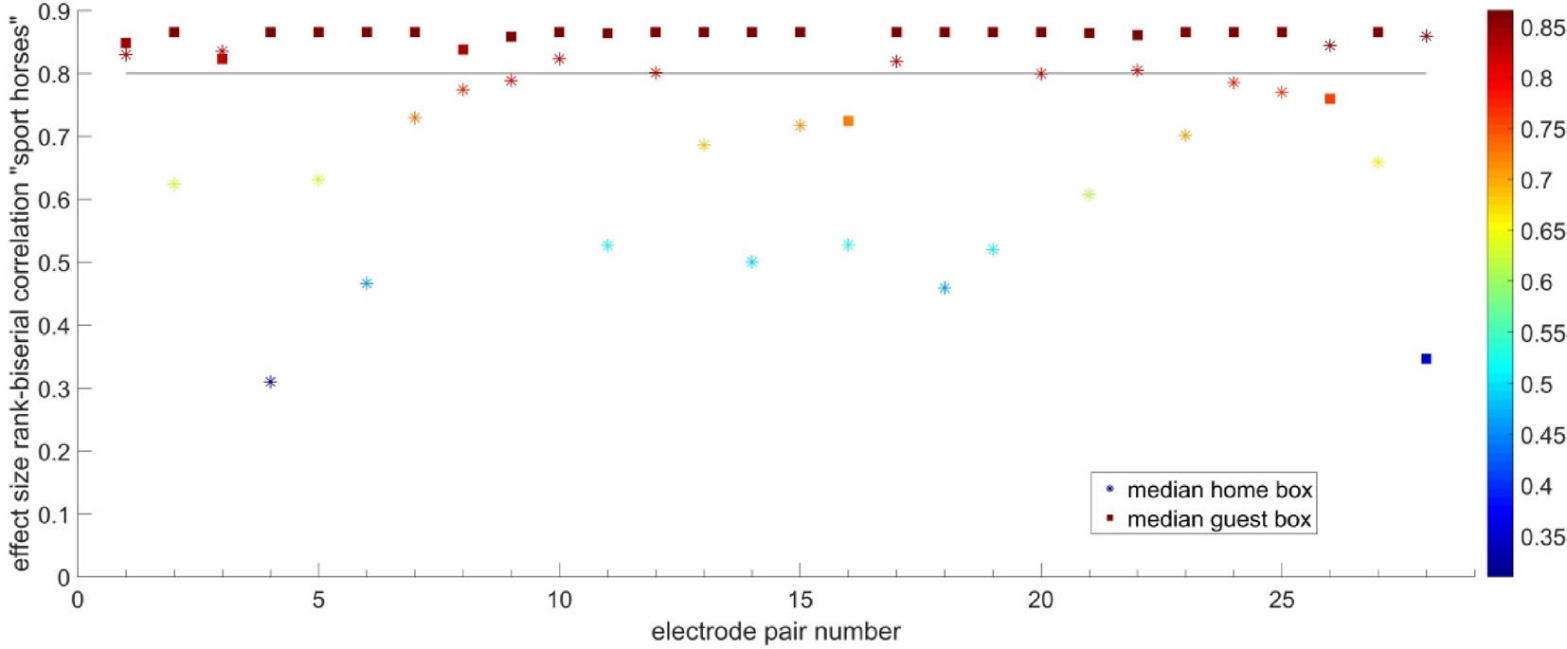
Effect size biserial rank correlation between electrode pairs for sport horses. Electrode pair 19 (occipital right and left, shows high correlation (threshold 0.8) in the guest box and low correlation for the home box.

**Fig. 5:**
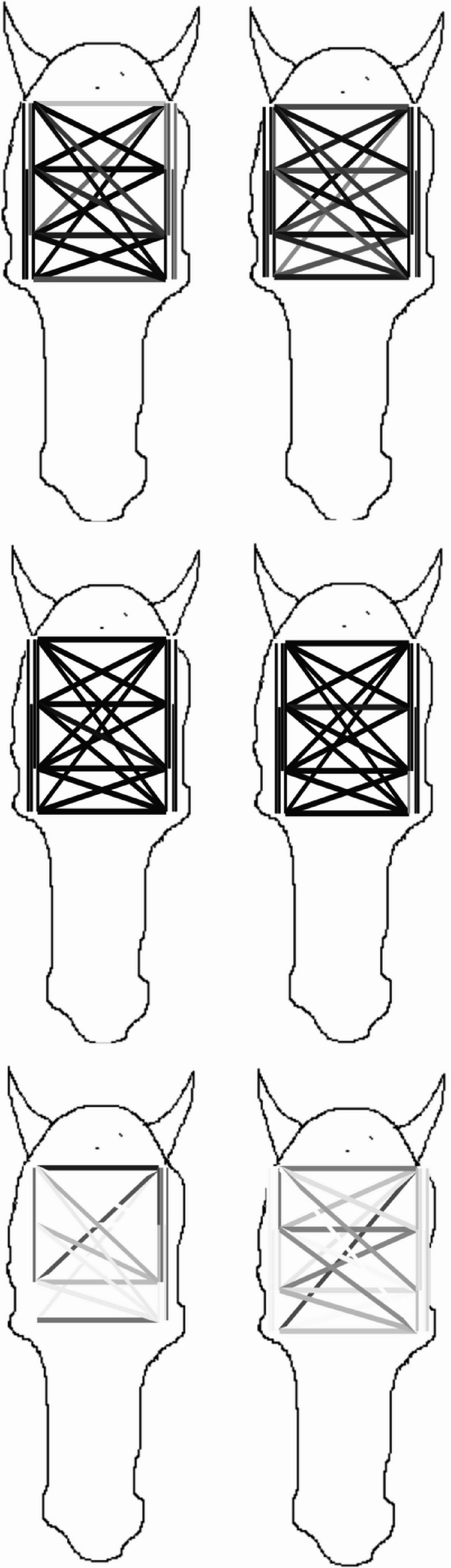
Median rank biserial correlation between electrode pairs, connected by lines. The darkness of the lines indicates the strength of the correlation. In the left column are non-sport horses, in the right column are sport horses. The first row represents the home box, the second row represents the guest box and the third row represents the difference between the home and guest box for both populations, respectively.

Regarding EEG analysis, only the delta band showed significant correlations between electrode pairs. The threshold to define a significant correlation was set to 0.8 (Figs. 3 and 4, correlation between 0 and 1, with 1 = maximal correlation). Out of the 28 different electrode pair correlations (Table 1), electrode pair 19 stood out as showing a difference between the home box and the guest box for both horse samples (sport horses and non-sport horses). Electrode pair 19 represents the correlation between electrodes 4 and 5, corresponding to the positions occipital right and occipital left. This position is characterized by the line between the ears in Fig. 5. Figure 5 displays the correlation values for electrode pairs in relation to the head with the values color coded where darker shades represent higher values (1 = black, 0 = white). The upper row shows the median values for the home box, the middle panel represents the guest box situation and the third panel shows the difference in median correlation between the home- and guest box. The left represents the non-sport horses, while the right row represents the sport horses. The median correlation is low in the home box and above 0.8 in the guest box (Figs. 3 and 4). Therefore, the distinct electrode pair for the correlation is interhemispheric. The correlation between electrode pairs is generally higher in the guest box compared to the home box.

### EEG analysis: coherence

**Fig. 6:**
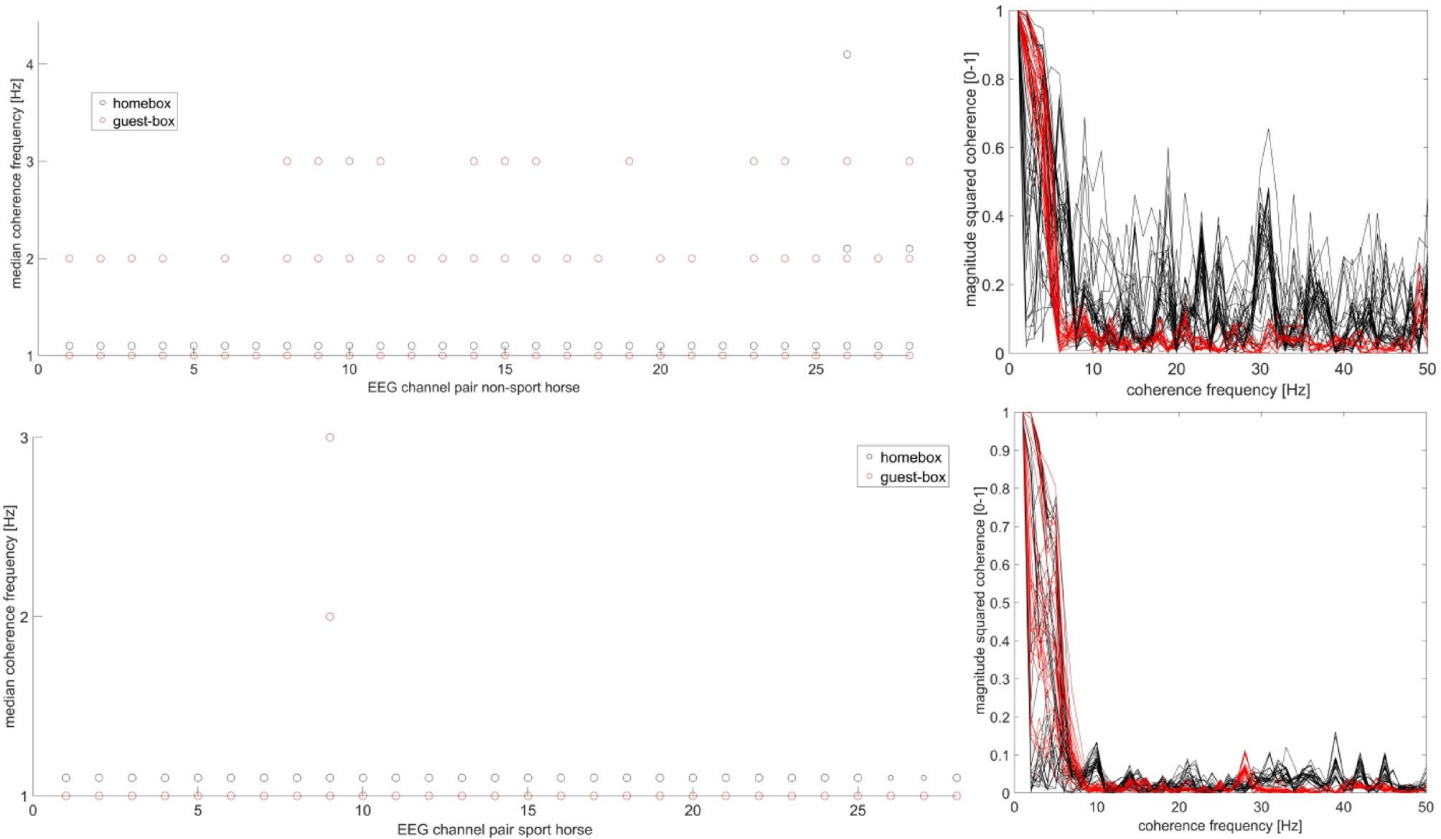
Magnitude squared coherence between EEG delta band and all other frequency bands. The upper two panels represent non-sport horses, while the lower row shows sport horses. The left two figures display the median coherence among 5 horses for home box (black) and guest box (red) for electrode pairs. A distinctive feature for the First Night Effect is coherence values at electrode pair 9 as the coherence profile changes similarly for both horse populations depending on the situation (home box/guest box). Electrode pair 9 represents the coherence between the right hemispheric occipital and temporal electrode position. The two figures on the right show magnitude squared coherence values (ranging from 0 to 1 with 1 indicating maximal coherence) for frequencies up to 50 Hz and all electrode pairs for all horses in the home box (black) and guest box (red). The coherence values in the home box exhibit a steeper decrease compared to the guest box, with a second increase around 5 Hz causing the red and black lines to intersect.

Delta-band coherence was only detectable within the delta band with no cross frequency coupling. Electrode pair 9 showed differences in coherence among home box and guest box for sport and non-sport horses, respectively (Fig. 6). The upper two panels in Fig. 6 represent the non-sport horses, while the lower row shows the sport horses. The left two figures display the median coherence among 5 horses for the home box and guest box, respectively. Coherence values at electrode pair 9 are a distinctive feature of the First Night Effect. The two figures on the right side show magnitude squared coherence values (ranging from 0 to 1, with 1 indicating maximal coherence) for frequencies up to 50 Hz and all electrode pairs for all horses in the home box (black) and guest box (red). The coherence values in the home box show a steeper decrease compared to the guest box with a second increase at approximately 5 Hz. The notable difference between the home box and guest box for non-sport horses is the high variance of coherence values in the home box which is not evident for sport horses in the lower right panel. The distinctive Electrode pair Nr 9 represents the coherence between electrode 2 (temporal right) and electrode 4 (occipital right). Hence, the distinctive electrode pair for coherence is intrahemispheric. All differentiating electrode pairs for the First Night Effect in horses are in an occipital position.

### EEG analysis: cross correlation, hemispheric activity

**Fig. 7:**
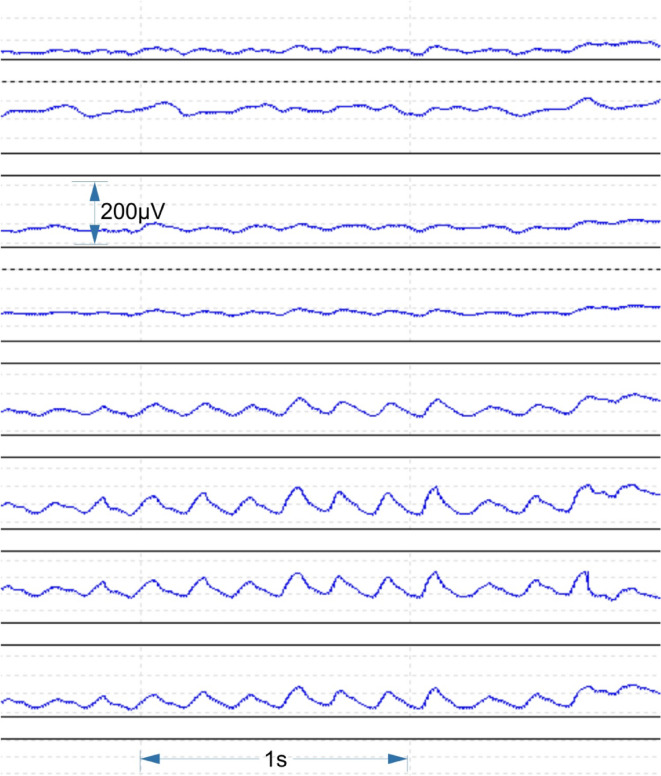
Unilateral theta waves in an individual non-sport horse during sleep. Channels from above: frontal right, temporal right, parietal right, occipital right, occipital left, parietal left, temporal left, frontal left.

**Fig. 8:**
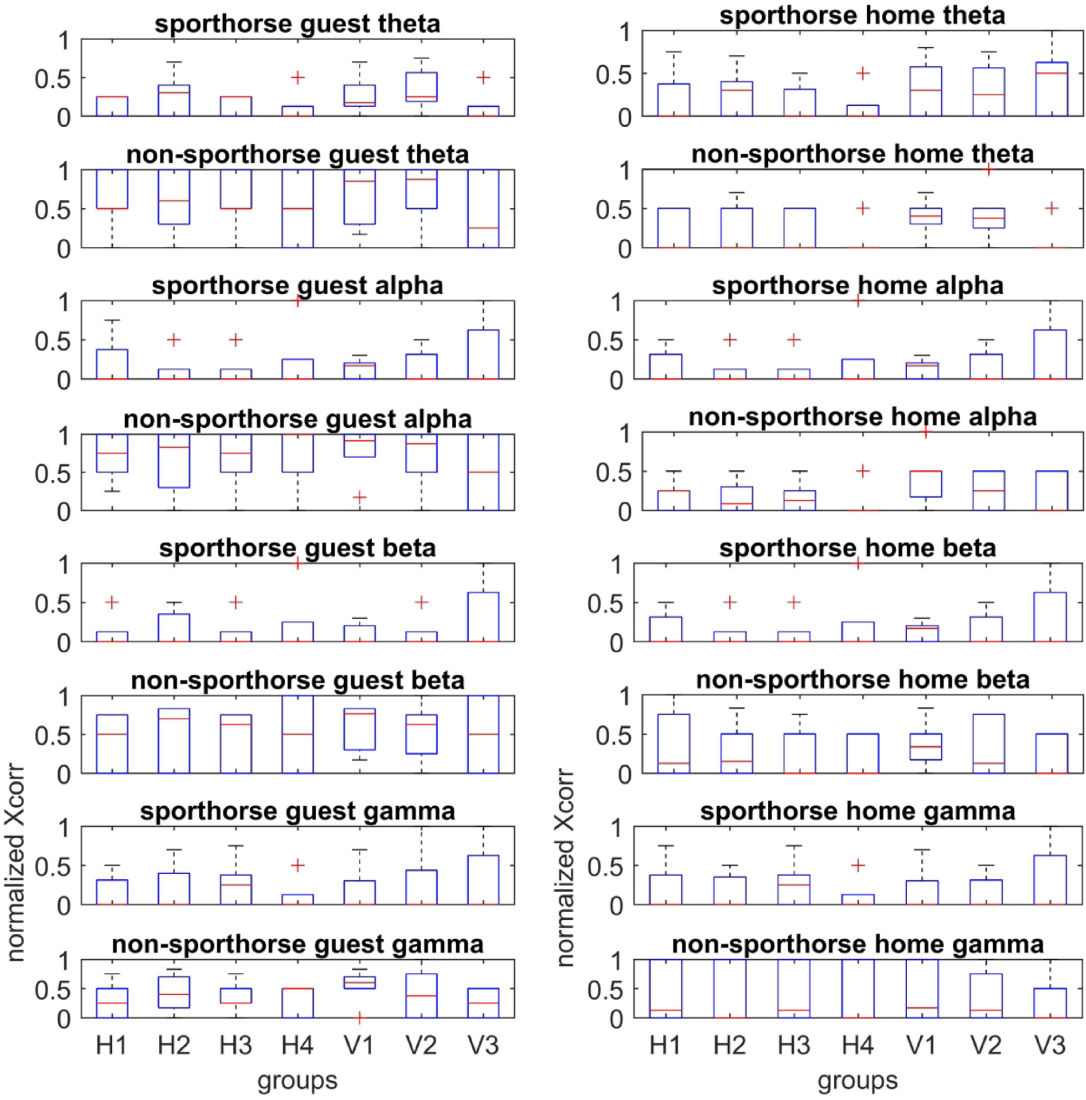
Boxplot of the normalized number of xcorr events for EEG groups H1 (horizontal) -V3 (vertical). A lower variability can be seen between guest box and home box for the sport horses compared to non-sport horses. There is a trend towards a lower number of normalized xcorr events in the home box in comparison to the guest box. Sport horses exhibit some degree of adaptation to the home box situation. The red line in the boxplots represents the median, the symbol “+” represents outlier values, the top edge of the box indicates 75^th^ percentile, and the bottom edge indicates the 25^th^ percentile.

A purely unihemispheric activity (Fig. 7) was ruled out with the xcorr analysis for the sleep phases selected for this dataset (drowsiness). Unihemispheric activity would have been characterized by a strong correlation for vertical electrode groups (V1-V3) instead of horizontal electrode groups (H1-H4). No preferred correlations for certain electrode groups (H1-V3) were found. Surprisingly, we found a very reduced variability in the number of normalized correlations between the guest box and the home box in case of the sport horses (Fig. 8). The difference values for the normalized number of xcorr events between home- and guest box are not distributed in the same way for all frequency bands and electrode groups. Statistically significant differences in the distribution of normalized xcorr difference amounts (difference of normalized xcorr amount between home- and guest box) between sport horses and non-sport horses are found for the theta band for electrode group H3 (*p* = 0.03) and V2 (*p* = 0.04). For the EEG band alpha, H2 and H3 (*p* = 0.03, respectively), V1 (0.004), V2 (0.03) and V3 (0.03), for the beta band with H1 (*p* = 0.01) and H2 (*p* = 0.004), V1 (0.005) and V2 (0.048) and for the gamma band with H2 (*p* = 0.004), H3 (0.03), H4 (0.03), V1 (0.004) and V3 (0.03) (Table 2). Each band has a unique pattern of xcorr representations but not with a simple underlying rule, like gamma representing long range groups. Gamma is the only band representing both long range differences (H4 and V3) and beta the only band representing both short range connections (V1 and H1). We excluded the delta band from analysis, because it showed high xcorr values for all electrode pairs and hence had no distinctive value. The xcorr analysis reveals a complex representation of the First Night Effect for all other EEG bands than delta. There is a tendency for the attenuation of xcorr difference values to the home box-situation within the sport horse population. Non-sport horses show a tendency for a lower amount of xcorr values in the home box rather than in the guest box (Fig. 8). Sport horses generally have a lower amount of xcorr values, even in the guest box.

An analysis for differences in the distribution of the occurrence of cross-correlations between the guest box and the non-guest box shows significant differences for the alpha EEG band and the horizontal electrode groups 1, 3 and 4 (ranksum test MATLAB, alpha 0.05: H1 *p* = 0.0195, H3 *p* = 0.039 and H4 *p* = 0.0281). This confirms the observed tendency or pattern that the distribution of xcorr events remains the same between home and guest box in the sport horses, while there are significant differences in interhemispheric EEG correlations in the alpha band in the non-sport horses.

## Discussion

The First Night Effect is well known among human travelers, who often experience compromised sleep quality in an unfamiliar place. Horses also frequently travel to sport events, breeding shows or veterinary facilities. In this study, we were able to demonstrate that horses do not exhibit a clear First Night Effect based on behavioral data (Video recordings), such as the amount of sleep. This is not surprising, as horses are flight animals and tend to conceal discomfort^20^. We did observe an indirect effect of the First Night Effect on behavior. We were surprised to find that sport horses had a longer duration of sleep in a lying position compared to non-sport horses. This effect was not evident in the guest box (see Fig. 2). Interpreting this finding in terms of welfare is challenging, as increased sleep duration in humans is typically associated with illness^16^. However, we were able to not only observe the First Night Effect in EEG recordings but also pinpoint changes in interhemispheric correlation and intrahemispheric coherence in the occipital brain region. In the guest box, coherence was detectable not only in the 1 Hz delta band but up to 3 Hz, unlike in the home box situation. This may indicate a transition from slow wave sleep to higher order delta band oscillations and a corresponding change in vigilance state, specifically sleep arousal. Previous research has shown that microarousals can influence hippocampo-cortical coherence and lead to shifts in the delta band^21^.

The interhemispheric correlation is generally higher in the guest box compared to the home box. This may be explained by the biphasic activity of cortical neurons, associated with the onset of slow waves in NREM sleep. The OFF phase of this bistable activity is proposed as a disintegrating force associated with fragmented consciousness and the onset of sleep^22^. When applied to the situation with horses in the guest box, the relatively high correlation in the occipital part of the brain might indicate extended consciousness or, in other words, a higher degree of alertness. In contrast, loss of consciousness is associated with a higher amount of slow wave sleep in the posterior part of the cortex^22^ and the decorrelation of neural networks.

Tamaki et al.^4^ found an asymmetry in hemispheric vigilance in humans, suggesting one hemisphere acts as a “night watch” in unfamiliar environments as a survival strategy. We also observed a predominantly unilateral coherence phenomenon during the First Night Effect. It is unclear whether this indicates unilateral sleep or asymmetric ensemble activation but we did find unilateral theta waves during sleep in individual horses (see Fig. 7).

Coherence in the 1 Hz range of the EEG spectrum was evident in both the home and guest boxes. In the guest box, there was an increased shift of delta band coherence within the 2–3 Hz range in an occipitotemporal position. Sport horses, which had some degree of habituation training for the First Night Effect (or more general for novel environments), displayed a home box coherence pattern similar to that of the guest box EEG in both sport and non-sport horses. These horses had relatively low coherence values and low jitter. The “First Night Effect adaptation training” for sport horses may have caused the coherence pattern to resemble that of a general guest box situation. The frequent changes in box location could have shifted sleep patterns towards the First Night Effect potentially negatively impacting the performance and well-being of sport horses.

A purely unihemispheric activity was ruled out for drowsiness sleep phases by a cross correlation analysis (xcorr, MATLAB). To better represent functional connectivity, we classified the suprathreshold correlation events into vertical (intrahemispheric) and horizontal (interhemispheric) groups, labeled V and H, respectively. The higher the number assigned, the greater the distance between the electrodes used for correlation measurement. In contrast to the delta band in coherence analysis, the xcorr analysis showed a decrease in the number of xcorr values, indicating the home box situation within the sport horse group. This “habituation” is characterized by EEG bands theta, alpha, beta and gamma, each showing a distinct pattern of group representation (vertical and horizontal EEG pair groups for different electrode distances). Overall, the sport horses exhibit significantly less variability in the number of correlation events in the EEG frequency bands between their own box and the external box compared to non-sport horses. Interpreting these results is challenging given the dynamic nature of sleep patterns in EEG recordings^23^. Notably, high correlations in the gamma band were observed in the home box, particularly for long-distance connections, while high correlations in the alpha band were found in the guest box. These findings may suggest changes in vigilance levels^24^. Research has linked high synchrony (low irreversibility) with slow wave sleep, and asynchronous activity (high irreversibility) with wakefulness^25^. The current study unveiled a complex picture, showcasing different dynamics of frequency bands across various cortical axes. Of particular interest is the reduced variability of correlations in sport horses indicating potential neural ensembles influencing sleep architecture beyond the occipital region, possibly during arousals. This area presents an exciting opportunity for future research, with implications for animal welfare and performance diagnostics.

All of these insights were achieved through a non-invasive EEG measurement technique without the need for a head cap. Understanding welfare-related or other EEG patterns potentially allow for individual diagnostics using EEG in the future.

Limitations of the study include poor video quality; better cameras could have led to a more precise behavioral analysis. Additionally, data loss occurred due to low battery power during night temperatures below − 15 ° degrees Celsius. Unfortunately, some very large horses also dismantled the camera system, resulting in further data loss. One horse even damaged the EEG system, despite the device being placed in a protective neck-pocket. A recording session was lost due to a lightning strike causing a power failure that affected the receiver and personal computer. On another occasion, the personal computer and attached receiver unit in the barn were shut down during the night, possibly due to playful stable cats. These incidents present a challenge to make the system more suitable for everyday use. The possible influence of gender or breed must be clarified in future studies.

In conclusion, there are significant variations in sleeping behavior and sleep-related EEG patterns within two populations of horses with different training, feeding, and general stable management routines. Despite the fact that the sport horse population had undergone some level of habituation training to the First Night Effect, it was still possible to identify anatomically defined EEG alterations associated with this phenomenon. These unique EEG patterns are primarily located in the occipital brain region and impact delta band modulations, including coherence and correlation. Cross correlations also indicate distinct habituation-like processes for the EEG bands ranging from theta to gamma. The delta band coherence decreases in response to the guest box situation while the theta to gamma bands for xcorr attenuate to the home box situation with relatively low amounts of xcorr events in sport horses, there may be some kind of functional dissociation between EEG bands in the sense that underlying neural networks may “habituate” or react in a totally different way with respect to sleeping in an unfamiliar environment.

Furthermore, we found that sport horses lie down for a longer duration than non-sport horses. While the pattern is still visible, the effect is compensated for at the statistical level by the First Night Effect, providing indirect evidence that sleeping behavior is impacted. Hence, in case of detection of the First Night Effect in horses, EEG data may be more sensitive than behavioral data. Coping strategies of flight animals can mask behavior, making EEG data potentially more objective for analyzing affective brain states. The key is to use a measurement technique that is inconspicuous for the test person (in this case the horse) and does not restrict movement or overall well-being. Otherwise, the measurement technology itself could falsify the EEG data. Through this study, we have demonstrated that the non-invasive and telemetric EEG measurement technique presented here is effective in visualizing even minor deviations in the EEG under everyday conditions without the need for a laboratory setting. This breakthrough opens up new possibilities for integrating behavioral and brain research with the potential to reduce the use of experimental animals in neuroscience. Additionally, there is potential for its application in medical and veterinary diagnostics.

## Data Availability

The datasets used and/or analysed during the current study available from the corresponding author on reasonable request.
